# Principles of Pathology and Practice of Medicine

**Published:** 1844-09

**Authors:** 


### Bibliographical Notices.

*Principles of Pathology and Practice of Medicine.* By John Mackintosh, M. D., Lecturer on the Practice of Physic, in Edinburgh, &c. Fourth American, from the last London Edition, with notes and additions. By Samuel George Morton, M. D. Philadelphia: Lindsay & Blakiston, 1844.

The high estimate placed upon this work by those best qualified to judge of its merit, is found in the fact, that while the press has been teeming with books upon the same subject, and while the publishers of medical periodicals are sending out excellent treatises upon medicine, in monthly and quarterly emissions, this large volume has gone through eight editions, four in England and an equal number in this country. The present comes to us with the additional value of Dr. Morten's notes and additions, which, he modestly tells us in his preface, would have been far more ably supplied by the hands of Dr. Mackintosh himself, had it not been for his "untimely and lamented death." We are not sure of this. Dr. Morton is an able and industrious man, and has given abundant evidence to the medical profession, of his capacity to do justice to any subject he may undertake to write upon. Moreover, the practice of physic in this country has some peculiarities. Indeed, in some respects, it is far in advance of the practice of the European schools. An English book on this subject, therefore, needs American notes, and we have no doubt that the additions, of which Dr. Morton speaks so humbly, add much to the value of the work before us.

As our space does not permit us to enter upon an examination of Dr. Mackintosh's practice, including as it does so many details, we content ourselves with recommending it to our readers. The practice of dentistry is essentially a department of medicine, and we are sure that the scientific of our profession will find pleasure and profit in the study of the work, under consideration.

